# The Influence of Sulphate Deposition on the Seasonal Variation of Peat Pore Water Methyl Hg in a Boreal Mire

**DOI:** 10.1371/journal.pone.0045547

**Published:** 2012-09-21

**Authors:** Inger Bergman, Kevin Bishop, Qiang Tu, Wolfgang Frech, Staffan Åkerblom, Mats Nilsson

**Affiliations:** 1 Department of Forest Ecology and Management, Swedish University of Agricultural Sciences, Umeå, Sweden; 2 Department of Aquatic Sciences and Assessment, Swedish University of Agricultural Sciences, Uppsala, Sweden; 3 Department of Earth Sciences, Uppsala University, Uppsala, Sweden; 4 Department of Analytical Chemistry, University of Umeå, Umeå, Sweden; Federal University of Rio de Janeiro, Brazil

## Abstract

In this paper we investigate the hypothesis that long-term sulphate (SO_4_^2−^) deposition has made peatlands a larger source of methyl mercury (MeHg) to remote boreal lakes. This was done on experimental plots at a boreal, low sedge mire where the effect of long-term addition of SO_4_^2−^ on peat pore water MeHg concentrations was observed weekly throughout the snow-free portion of 1999. The additions of SO_4_^2−^ started in 1995. The seasonal mean of the pore water MeHg concentrations on the plots with 17 kg ha^−1^ yr^−1^ of sulphur (S) addition (1.3±0.08 ng L^−1^, SE; n = 44) was significantly (p<0.0001) higher than the mean MeHg concentration on the plots with 3 kg ha^−1^ yr^−1^ of ambient S deposition (0.6±0.02 ng L^−1^, SE; n = 44). The temporal variation in pore water MeHg concentrations during the snow free season was larger in the S-addition plots, with an amplitude of >2 ng L^−1^ compared to +/−0.5 ng L^−1^ in the ambient S deposition plots. The concentrations of pore water MeHg in the S-addition plots were positively correlated (r^2^ = 0.21; p = 0.001) to the groundwater level, with the lowest concentrations of MeHg during the period with the lowest groundwater levels. The pore water MeHg concentrations were not correlated to total Hg, DOC concentration or pH. The results from this study indicate that the persistently higher pore water concentrations of MeHg in the S-addition plots are caused by the long-term additions of SO_4_^2−^ to the mire surface. Since these waters are an important source of runoff, the results support the hypothesis that SO_4_^2−^ deposition has increased the contribution of peatlands to MeHg in downstream aquatic systems. This would mean that the increased deposition of SO_4_^2−^ in acid rain has contributed to the modern increase in the MeHg burdens of remote lakes hydrologically connected to peatlands.

## Introduction

Historically, human activities have caused large emissions of mercury (Hg) to the atmosphere. This has contributed to widespread Hg pollution and increased concentrations in the biota e.g. fish. Deposition of Hg in the boreal region modelled by EMEP [1] show relatively low rates in the Nordic countries, deposition rates that also are verified by moss monitoring [2]. The concentrations of Hg in fish are higher, though, than in other regions with similar or even higher deposition rates [3]. One key to the difference between Hg deposition and the high degree of bioaccumulation is one particular species of Hg, methyl mercury (MeHg) that is most prone to bioaccumulation [4]–[6]. The cycling of MeHg in boreal catchments is complex and involves numerous biogeochemical and physical controls. Therefore, it is important to elucidate the impact of these factors on MeHg production.

Important sources of MeHg loadings to lakes are direct precipitation [7], [8], run-off from wetlands [9]–[11], and in-lake methylation [12]–[14]. Mass-balance calculations, however have revealed that often neither precipitation, nor in-lake methylation can account for the total amount of MeHg in the catchment runoff [15] or in lakes [16]–[18]. The runoff of MeHg from catchments and the levels found in fish can vary considerably between different catchments with similar levels of atmospheric deposition [5], [19]. This suggests that the MeHg species is produced within the catchments.

Boreal wetlands have been identified as an important source of MeHg to lakes [16], [20]–[23] though there is considerable variability in the strength of these hotspots across the landscape [24]–[26]. One factor affecting the significance of wetland sources can be the proportion of mires [27] or riparian zones [28], [29] in the catchment. While wetlands generally are considered sources of MeHg one alder swamp have actually been found to be a sink of MeHg [25]. Another possible factor in the catchment production of MeHg is sulphate (SO_4_^2−^) deposition which stimulates the production of MeHg [9], [12], [30].

The importance of sulphate-reducing bacteria (SRB) for methylation of Hg is well-documented [12], [31], [32]. The possibility that SO_4_^2−^ in ”acid-rain” has enhanced in-lake methylation was suggested by Gilmour and colleagues [12], [33]. A number of studies now suggest that the availability of SO_4_^2−^ for SRB is a major factor regulating the concentration of MeHg in mire pore water. Branfireun et al. [30] suggested SO_4_^2−^ reduction in the anoxic zone of mires and riparian wetlands as the functional explanation for the increased concentrations of MeHg in runoff from boreal wetlands. In that study addition of SO_4_^2−^, corresponding to 28 kg SO_4_^2−^ ha^−1^, to a boreal peatland increased the pore-water concentrations of MeHg from 2 to 4 ng L^−1^ within 24 hours. The MeHg concentrations, however, returned to ambient levels after 5 days. In constructed wetlands, Harmon et al. [34] found that SO_4_^2−^ amendments gave significantly higher MeHg pore water concentrations over the course of one year. In a mescosm experiment, Mitchell et al. [35] found that SO_4_^2−^ additions significantly increased MeHg pore water concentrations, while additions of different carbon substrates alone did not have an effect. Combined SO_4_^2−^ and carbon (C) additions, however, gave the largest increases, providing an explanation of why hotspots of MeHg appear in mires where there are inputs of both C and SO_4_^2−^ [23].

An experiment to simulate the effect of atmospheric SO_4_^2−^ at the ecosystem scale by sprinkling SO_4_^2−^ solutions systematically across a mire for one snow-free season also found a significant influence on MeHg, both in pore water and in the runoff from the mire [9]. SO_4_^2−^ additions in that experiment were made on five occasions, and the effects on pore water concentrations varied considerably. This may have to do with the relatively short term additions of SO_4_^2−^ to the system, a feature common to most other studies on how SO_4_^2−^ influences MeHg in mires.

Pore [36] and run off [9] water from wetlands responded to experimentally elevated sulphur (S) deposition with an increased potential to produce MeHg. Branfireun et al. [36] found a tripling of peat pore water MeHg in plots exposed to two years of elevated S deposition (20 kg S ha^−1^ yr^−1^) compared to plots with the ambient deposition in N. Sweden (3 kg S ha^−1^ yr^−1^), five weeks after the most recent experimental SO_4_^2−^ amendment had been made. An almost equally large increase (2.4 times) in stream water MeHg concentrations were also found after experimental additions of SO_4_^2−^ of an experimental wetland in northeastern Minnesota [9]. Such long-term effects would greatly increase the potential significance of S deposition for MeHg-loading to the aquatic ecosystem.

Observation of elevated MeHg at a single point in time is not, however, sufficient to prove a persistent effect since other factors are also important for the regulation of SO_4_^2−^ reduction (and hence any sustained effect of SO_4_^2−^). These factors include the occurrence of competing e^–^acceptors e.g. oxygen which is mainly determined by the prevalence of saturated conditions [37], [38], availability of easily degradable C for the SRB [35], [39], [40] and temperature [41]. To investigate the importance of these factors for the persistence and magnitude of the “acid-rain” effect, we studied the differences in temporal variation of peat pore-water MeHg between plots exposed to ambient (ca 3 kg S ha^−1^ yr^−1^) and five years of enhanced SO_4_^2−^ deposition (20 kg S ha^−1^ yr^−1^) in a low sedge mire in northern Sweden during one snow-free period of five months duration. On three occasions we sampled these plots at four depths to get a deeper profile of pore water MeHg. Our main hypothesis is that elevated pore-water MeHg concentrations are maintained by an increased pool of S (due to enhanced deposition) which can be recycled at the mire surface through reduction of SO_4_^2−^ (to e.g. HS^-^) and subsequent re-oxidation of reduced S species to SO_4_^2−^. Since we are interested in ascertaining the effect of this process on MeHg loading to lakes and their biota, we have sampled the uppermost ten centimetres below the water table every week during the study period, regardless of the water table depth relative to the mire surface. This is the water most likely to compose the runoff from wetlands to downstream lakes [42].

## Materials and Methods

### Field Experimental Setup and Sampling

The pore water samples were collected from a low sedge mire, Degerö Stormyr (Lat. 64°11′N, Long. 19°33′E, altitude 270 m a s l) situated 70 km from the coast of the Gulf of Bothnia, Sweden. The experimental site is part of the Vindeln Research Forests, Swedish University of Agricultural Sciences. The experimental set-up was established in 1995 and is based on the manipulation of 20, 4 m^2^ plots, each of which are isolated from the surroundings by a 0.5 m deep plastic frame inserted to a depth of 0.4 m in the peat. Together with one extra control plot without plastic frames, there were a total of 21 experimental plots. In this study we utilised four of these plots, two receiving ambient levels of S (3 kg S ha^−1^ yr^−1^) from the precipitation, and two plots receiving experimental additions, applied at five occasions during the snow free period, which together with the ambient input equals 20 kg S ha^−1^ yr^−1^. A thorough description of the experiment is given in Granberg et al. [43] and Wiedermann et al. [44].

Pore water from these plots was sampled every week between May 26^th^ and October 20^th^ in 1999, 0–10 cm beneath the groundwater level (GWL) at the time of sampling. In addition, pore water was sampled from 10–20 cm, 20–30 cm, and 30–40 cm beneath the GWL on June 23^rd^, July 28^th^, and August 25^th^ 1999. The actual GWL was measured at 5 evenly distributed points within each plot. Runoff water in the stream draining the mire was also sampled each week, starting June 23^rd^.

Subsamples for dissolved organic carbon (DOC) were kept cool and in the dark before analysis within one month after sampling. The subsamples for anions were preserved by freezing, and the subsamples for cation analysis were preserved with suprapur sulphuric acid. The anions and cations were all analyzed within six months of sampling.

The DOC was determined by analytical combustion (Shimadzu, DOC-500). Pore water concentration of SO_4_^2−^ was determined by suppressed ion chromatography (Shimadzu, UV detector SPD-6A) using 0.5 mM sulfosalicyl acid as the mobile phase. After filtration, pH was determined in all pore water samples. Total S, Hg_tot_ and MeHg in the solid peat were determined on freeze-dried and ground peat samples taken at 0–5 cm, 5–10 cm, 10–20 cm, and 20–40 cm beneath the surface of the sphagnum moss. The solid samples were collected on June 24^th^ 1999. For total S, the samples were digested with HNO_3_+ HClO_4_ and thereafter analysed using Inductive Coupled Plasma – Atomic Emission Spectrometer (ICP – AES, Perkin Elmer).

#### Pore water sampling procedure

Pore water samples were collected with a 70 cm long, custom-made, Teflon sampler (13/6 mm, outer/inner diameter) equipped with 4 holes (3 mm diameter) for every 1 cm of the lower 10 cm i.e. a total of 40 holes. At the end of the sampler there was a removable conical tip to facilitate penetration of the peat surface. The sampler was connected to a 500 mL transfer bottle (Pyrex^©^) by Teflon tubing (8/6 mm, outer/inner diameter), and the transfer bottle was connected to a custom-made, portable vacuum pump. Even though the air flows in the direction from the transfer bottle to the vacuum pump, diffusion of Hg originating from the pump constitute a mass transfer in the opposite direction that potentially might contaminate the pore water samples. To avoid potential contamination from the vacuum pump, gold traps were installed between the vacuum pump and the transfer bottle or the filter holder.

Samples were collected by inserting the sampler 10 cm below the actual GWL and then pumping the pore water into the transfer bottle. Approximately 100 mL of water was collected from 5 evenly distributed spots within each experimental plot to create a pooled sample of 500 ml. Approximately 150 mL of the pooled sample was then filtered through an acid washed membrane filter (45 mm diameter, 0.45 µ, cellulose nitrate 100%, sterile, Millipore®) using a 500 mL filter holder (Nalgene®). Filter holders were connected to a manifold which in turn was connected to a portable vacuum pump (Prenart equipment ApS, Frederiksberg, Denmark). This set-up allowed four samples to be filtered simultaneously. Immediately after filtration, each sample was transferred to a Teflon bottle (Nalgene®), put in a plastic bag, and then kept cool on dry ice in the dark. For stream water, a grab sample was taken directly in a similar Teflon bottle without filtration due to the low content of particulate matter. The samples were stored in the laboratory at +4°C until analysis. The MeHg and total Hg in the pore water were always determined within a maximum of five days.

Separate tests before the start of the investigation showed negligible losses of MeHg during five days of storage in the dark and at 4 ^o^C. In an earlier study [45] the stability of spiked (1.0 ng/L) MeHg in river water was determined and only minor losses of MeHg during the storage of samples was found. After 15 days storage in Teflon containers without additions of preservation reagents the mean concentration of MeHg was decreased by 6%. (The concentration of MeHg originally present was 0.35 ng L^−1^ and 4.1 ng Hg^2+^ L^−1^; TOC was 13 mg L^−1^ and pH was 5). Prior to each sampling occasion, the Teflon bottles, transfer bottles, filtering units, filters, Teflon tubing and the Teflon sampler were acid washed with HCl (p.a. quality, pH = 2) over night at 70°C, rinsed three times with Millipore water and thereafter kept in plastic bags prior to the sampling. Plastic gloves were always used during washing and sampling, or when otherwise handling the equipment.

### Determination of Hg_tot_ and MeHg in Peat, Pore Water and Stream Samples

Chemicals were of analytical-reagent grade unless indicated otherwise. All standards and solutions were prepared using Millipore deionized water (Millipore, Bedford, MD, USA).

### Digestion and Determination of Hg_tot_

#### Peat samples

Some 200 mg of peat (wet weight) were weighed and placed into Teflon tubes. Then 4 ml of HNO_3_ and 4 drops of HCl were added to samples that were left overnight on a clean bench. After this 10 drops of H_2_O_2_ were added and samples were subsequently heated to 80°C for 8 hours. 4 ml of H_2_O were added and after centrifugation the clear solution was used for quantification of Hg. The Hg species in the samples were reduced using SnCl (30%) to Hg^0^ that was purged onto gold traps using argon gas. The Hg adsorbed to gold traps was thermally desorbed followed by analysis using cold vapour atomic absorption spectrometry (CVAAS) at 254 nm (FIMS, Bodenseewerk Perkin Elmer, Überlingen, Germany) [46]. All samples were analysed at least two times. Tests with addition of 50 and 100 µg HgCl L^−1^ to one of the samples before acid treatment gave recoveries of 100.9 and 98.7%. Certified reference material (Light sandy soil, BCR 142 with a certified content of Hg of 67±11 ng g^−1^) was analysed in the series and gave a value of 74.0±11.0 ng g^−1^ (n = 3). The SO_4_^2−^ salt used in the field application treatments was analyzed for Hg_tot_ which was found to be below the detection limit of 40 pg Hg g^−1^ (3σ criterion [47]).

#### Pore and stream water

Pore water (30 mL), HNO_3_ (3 mL ultra pure), and HCl (0.5 mL ultra pure) were added to quartz flasks, which thereafter were subjected to UV radiation for seven hours. After digestion, 5 mL of a solution containing 30% SnCl and 5% H_2_SO_4_ was added to the sample. The Hg^0^ formed was thereafter removed during 10 minutes under a constant flow of Argon. Hg^0^ was amalgamated on a gold trap and thermally desorbed and detected by CVAAS as described above [46].

### Extraction and Derivatization Procedure for the Determination of MeHg

#### Pore water and streamwater

A modified *in situ* ethylation method using tetraethylborate [48], [49] was employed for analysis of MeHg concentration in pore water samples [50]. Derivatized Hg species, trapped to Tenax (Supelco, Bellefonte, PA, USA) columns was thermo-desorbed at 200°C and injected into the GC for determination by Microwave Induced Plasma – Atomic Emission Spectrometer (MIP-AES) [51].

### Peat Samples

A solvent extraction (0.03 M CuSO_4_ (3 mL), 0.38 M KBr (3 mL), and CH_2_Cl_2_ (5 mL)) method [52] followed by ethylation using tetraethylborate [49] and GC separation and MIP-AES detection were used for analysis of the total MeHg content in peat samples [51]. The absolute detection limit for MeHg determined with the *in situ* ethylation method was 1.7 pg L^−1^ as Hg based on three times the relative standard deviation of repeated blank measurements [47]. For quality assurance of the *in situ* ethylation procedure standard additions of MeHg were performed to determine recoveries. These were found to be 0.90–1.05 for an increase of MeHg concentrations in the range of 1 ng L^−1^. The ethylation method provided accurate results (within 2% of the certified value) for MeHg when analysing certified reference materials (Tort 2; Dolt 2) and it provided good agreement with results for MeHg in brackish water 0.12±0.009 ng/L compared to 0.1±0.002 ng L^−1^ for flow injection-liquid chromatography-CVAAS. For the comparison three determinations were performed with each method. The precision of the *in situ* ethylation method was typically better than 10% for the pore water samples. Normally one analysis was performed for each pore water sample.

### Statistics

To compare the pore water MeHg concentrations between the plots with 20 kg S ha^−1^ y^−1^ (HighS) and 3 kg S ha^−1^ y^−1^ (LowS) plots, General Linear Model (GLM) statistical analysis (SYSTAT inc.) was run with treatment (HighS, LowS) as the category variable, groundwater level (GWL) as the independent variable and MeHg concentration as a dependent variable. The GLM was also used to compare the pore water MeHg concentrations at different depths, with treatment, depth, and date as category variables, while the concentration of MeHg was the dependent variable.

Statistical comparison of the concentration of MeHg, Hg_tot_, and S_tot_ (dependent variables) in the peat organic matter between the HighS and LowS plots was also made using GLM. Here, the category variables used were: treatment (HighS or LowS) or depth (0–5 cm, 5–10 cm, 10–20 cm, 20–40 cm). GLM was also used for comparing pore water pH (dependent variable) between the HighS and LowS plots (category variable). All pairwise comparisons within each variable were made using Tukey’s test.

## Results

**Figure 1:**
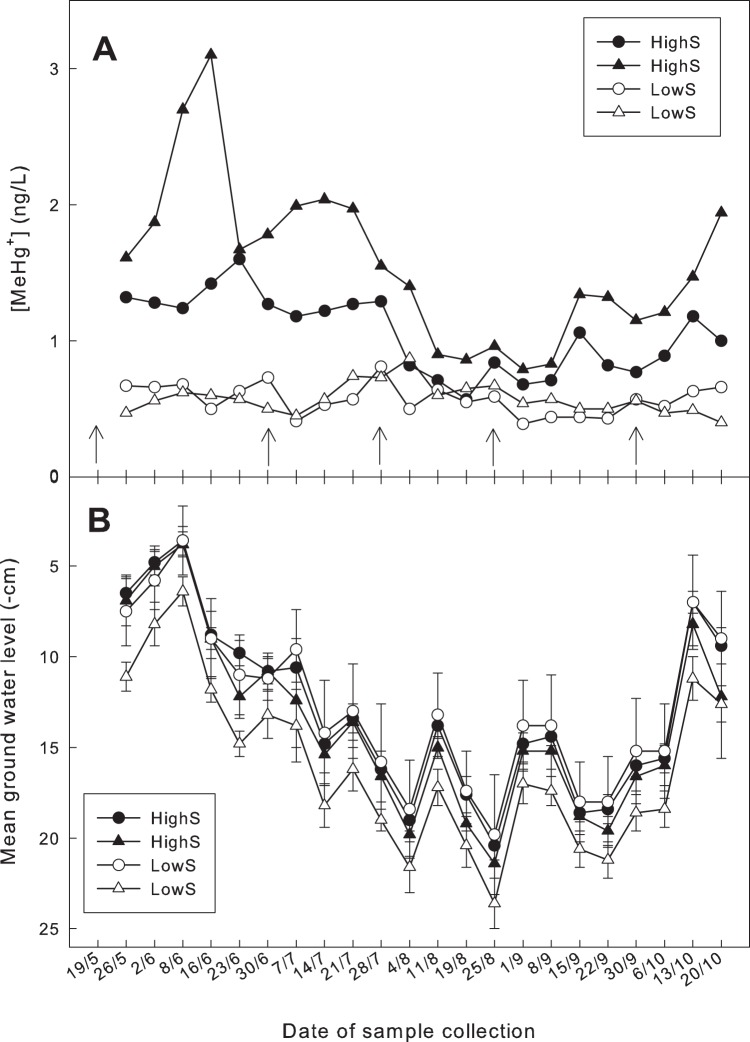
Weakly variations in A: concentrations of MeHg in pore water collected 10 cm below the groundwater surface at the time of sampling from HighS plots (filled symbols) or LowS plots (open symbols), as well as in the main stream draining the mire where the study was conducted (shaded squares). B: mean groundwater level from the HighS plots (filled symbols) or LowS plots (open symbols). The bars indicate SE. The arrows in A refer to the date on which the plots were fertilised with SO_4_^2−^.

The concentrations of pore water MeHg were always measured in the top ten centimetres below the water table. The depth of the mire water table relative to the mire surface varied ∼15–20 cm during the measurement period. The variation in pore water MeHg concentrations may therefore result from both seasonal variation in production/consumption of MeHg at each depth as well as differences in production/consumption between depths. The seasonal mean of the pore water MeHg concentrations (1.3 ng L^−1^±0.08, SE; n = 42) from the plots receiving additional SO_4_^2−^ (HighS) was significantly (p<0.0001) higher than the MeHg concentrations (0.6 ng L^−1^±0.02,SE; n = 42) in the plots receiving ambient levels of SO_4_^2−^ (Fig. 1A). A pronounced, systematic seasonal pattern in MeHg concentrations was found in the HighS plots, but not in the plots receiving ambient S deposition (3 kg ha^−1^, LowS). Generally for both HighS plots the pore water MeHg level was highest from the beginning of the summer until late July. Thereafter the MeHg concentrations decreased to the lowest levels from the middle of August to the beginning of September. The pore water concentrations of MeHg then increased again (Fig. 1A). In the LowS plots, the amplitude of MeHg variation was lower (a standard deviation of 0.11 ng L^−1^, with a range of 0.48 ng L^−1^ between the highest and the lowest values) than in the High S plots which had a standard deviation of 0.53 ng L^−1^ and a range of 2.55 ng L^−1^ (Fig. 1A).

The concentrations of pore water MeHg in the HighS plots were positively correlated (r^2^ = 0.21; p = 0.001) to the GWL at the time of sampling, with the lowest concentrations of MeHg during the period with lowest GWL (Fig. 1A, B). For the LowS plots there was no correlation between MeHg concentrations and the local groundwater table (p = 0.87) (Fig. 1A,B).

**Figure 2:**
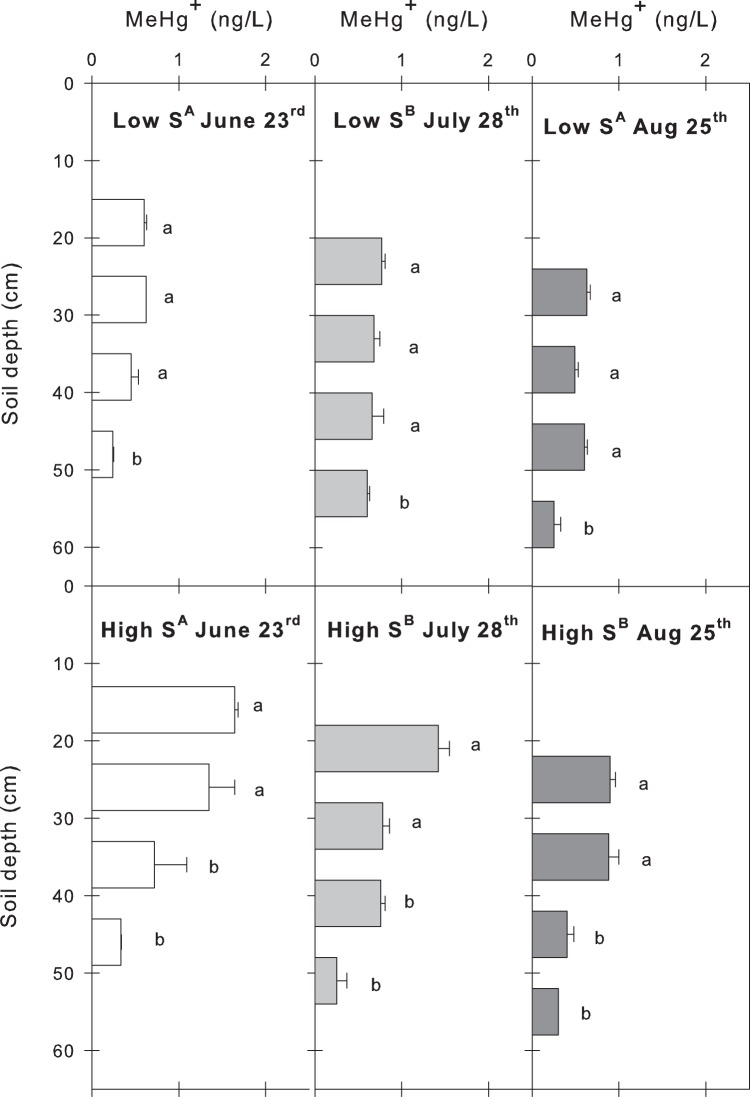
Depth profiles of MeHg pore water concentrations sampled 0–10 cm, 10–20 cm, 20–30 cm and 30–40 cm below the groundwater level at the LowS (upper panel) and HighS (lower panel) plots. The vertical axis refers to the actual peat depths at which the water was sampled. Each bar represents the mean value (±SE) of MeHg from two plots for the particular treatment. For each treatment (LowS or HighS), significant differences (p<0.05) in the average pore water MeHg concentration between the sampling dates are designated by different upper case letters. Different lower case letters indicate significant differences (p<0.05) in MeHg concentrations between depths for each treatment and sampling date.

The mean pore water concentrations of MeHg decreased with depth from the current GWL down to 40 cm below, for both the LowS and HighS plots (i.e. samples were taken at a maximum depth of 60 cm below the vegetation surface; Fig. 2). The decrease in MeHg concentrations with depth were more pronounced at the plots with enhanced SO_4_^2−^ deposition than those with ambient SO_4_^2−^ deposition levels (Fig. 2). For the LowS plots the MeHg concentrations at the deepest sampling depth (30–40 cm) were significantly lower than the three more superficial sampling depths for all three dates of sampling (Fig. 2, upper panel). In the HighS plots the pore water concentrations of MeHg were significantly (p<0.0001) higher in the two upper sampling depths compared to the two lower sampling depths (Fig. 2, lower panel).

The MeHg concentrations in the stream runoff (Fig. 1A, mean 0.6 ng L^−1^±0.06, SE; n = 13) were very similar to the concentrations measured just below the fluctuating groundwater surface on the ambient deposition plots (0.6 ng L^−1^±0.02, SE; n = 42) both with regards to absolute concentrations and variability over the course of the year.

The pore water concentrations of Hg_tot_ varied between 0.3 ng L^−1^ (detection limit) and 30 ng L^−1^ during the sampling period. No correlations (p>0.1) between the concentrations of MeHg and total Hg were found in any of the four plots, and there were no differences in these concentrations between the HighS plots and the LowS plots (p>0.1).

**Figure 3:**
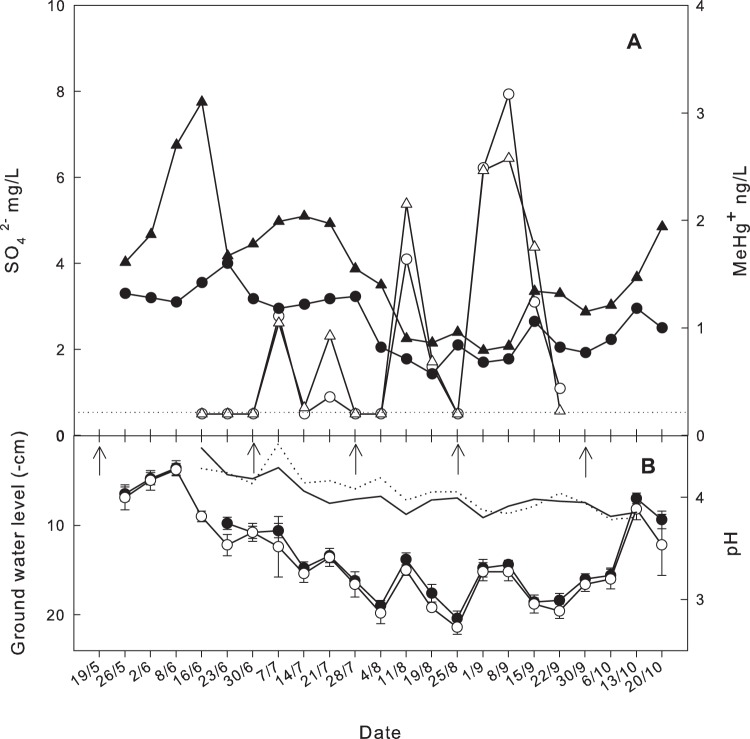
Weakly fluctuations of A: SO_4_^2−^ (filled symbols) and pore water MeHg (open symbols) concentrations, and B: the groundwater level between June 16^th^ and September 22^nd^ for both HighS plots. The concentration of SO_4_^2−^ in the LowS plots was below the detection limit during the sampling period. The dotted line in A indicates the detection limit for SO_4_^2−^ (0.5 mg L^−1^). The pH in the pore water for the two HighS plots are shown in panel B by the two lines without labels. The arrows in B refer to the date on which the plots were fertilised with SO_4_^2−^.

The pore water concentrations of SO_4_^2−^ were analysed for the period between June 16^th^ and September 22^nd^. Until the beginning of August, during the period with the highest pore water MeHg, the concentrations of SO_4_^2−^ in the HighS plots were very low, often below the detection limit (0.5 mg SO_4_^2−^ L^−1^) (Fig. 3A). However, during the period when the pore water MeHg concentrations in the HighS plots were lowest (and thus closest to the more stable MeHg concentrations in the LowS plots), the highest SO_4_^2−^ concentrations in the HighS plots were measured. The SO_4_^2−^ concentration in the HighS plots reached their highest levels when the GWL’s were deepest, between 15 and 21 cm below the mire surface (Fig. 3A, B). In the LowS plots the pore water SO_4_^2−^ concentrations were always below the detection limit (data not shown).

A seasonal trend in pH was observed in the HighS plots (Fig. 3B) with a slow decrease in pH from 4.4 (±0.1 SE, n = 2) to 3.8 (±0.02 SE, n = 2) between June 16^th^ and October 13^th^, (Fig. 3B). The corresponding pH values in the pore water samples from the LowS plots were 4.0 (±0.02 SE, n = 2), and 3.9 (±0.02 SE, n = 2), respectively (data not shown). The seasonal mean pH in the pore water from the HighS plots (4.0±0.2 SE, n = 36) was significantly different (p = 0.002; N = 72) from the seasonal mean pH in the LowS plots (3.9±0.1 SE, n = 36).

**Figure 4:**
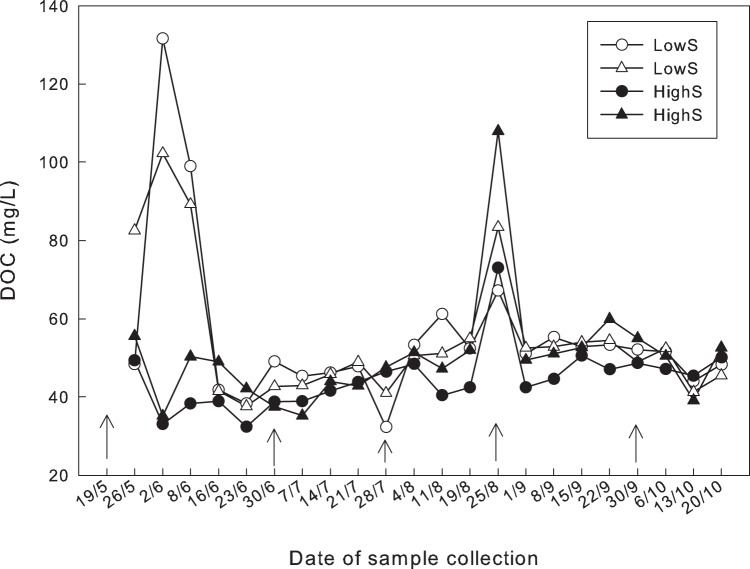
Weakly variation of dissolved organic matter (DOC) for HighS plots (filled symbols) and for LowS plots (open symbols). The arrows refer to the date on which the plots were fertilised with SO_4_^2−^.

There was a small but steady increase in the concentrations of DOC during the sampling period except in the beginning of June when the DOC concentrations in the LowS plots increased to approximately twice the value of the High S plots (Fig. 4). The highly elevated DOC values on August 25^th^ coincided with the deepest sampling depth for each of the plots (Fig. 1B).

The amount of S in the peat organic matter ranged between 0.05% and 0.16% (Table 1). There was no significant correlation between total S content in the peat and pore water MeHg. There was no significant difference in the concentration of MeHg in the peat between the LowS and the HighS plots (Table 1). However, in one of the HighS plots the concentration of MeHg in the organic matter was substantially larger compared with the LowS plots (Table 1).

**Table 1:** Content of total S (S_tot_ %), Hg_tot_ (ng g^−1^), and MeHg (ng g^−1^) in the organic matter at different depths from the two plots receiving 3 kg S ha^−1^ yr^−1^ (LowS) or 20 kg S ha^−1^ yr^−1^ (HighS).

Analysis	LowS	LowS	HighS	HighS
S_tot_ (%)				
0–5 cm	0.05	0.06	0.06	0.07
5–10 cm	0.05	0.06	0.05	0.07
10–20 cm	0.07	0.09	0.10	0.12
20–40 cm	0.10	0.16	0.10	0.12
Hg_tot_ (ng g^−1^, S.E., n = 2 or 3)				
0–5 cm	22(6.5)	27(14)	50(8.7)	64(4.0)
5–10 cm	68(15)	45(1.3)	59(3.2)	63(2.3)
10–20 cm	65(17)	68(2.2)	66(3.2)	57(0.9)
20–40 cm	79(5.0)	67(0.6)	89(8.6)	80(0.7)
MeHg (ng g^−1^)				
0–5 cm	0.17	0.30	0.20	0.76
5–10 cm	0.21	0.22	0.18	3.70
10–20 cm	0.26	0.34	0.42	1.90
20–40 cm	0.20	0.93	0.18	0.26

The solid samples were collected on June 24^th^ 1999.

The concentrations of total Hg were several orders of magnitude higher than the concentration of MeHg in the peat organic matter (Table 1). In the LowS plots there were significantly (p<0.001) lower concentrations of Hg_tot_ in the upper peat (0–5 cm) compared to the lower depths (Table 1). The concentrations of Hg_tot_ in the HighS plots were similar down to 20 cm peat depth, and thereafter the Hg_tot_ concentrations increased significantly (p = 0.026) (Table 1). The peat organic matter Hg_tot_ concentrations were significantly (p<0.001) different between the HighS and the LowS plots. The amount of Hg-tot in the solid phase of the upper 5 cm was significantly (p = 0.001) higher in the HighS plots compared to the LowS plots. The salt used (Na_2_SO_4_) to experimentally increase the load of S to the plots would cause an increase of 40 pg assuming that all Hg in the salt added over five years is retained. This amount is three orders of magnitude less than the difference between the Hg in the upper 5 cm of the HighS plots relative to the LowS plots. So the content of Hg_tot_ in the HighS plots do not emanate from the Na_2_SO_4_ additions.

## Discussion

Boreal mires have earlier been identified as an important source of MeHg loading to surface waters [20], [21], [27], [53]. There is increasing awareness that SO_4_^2−^ is an important control on the strength of mires as a MeHg source. A number of short term studies have seen this after one to half a dozen SO_4_^2−^ amendments, during the course of up to one year [9], [30], [35]. The potential for chronic (multiple years) of SO_4_^2−^ addition to increase the mire pore water MeHg concentration was demonstrated by Branfireun et al. [36] based on data from one point in time during 1997 from the same experimental site used in this study. Our results from the entire snow-free period of 1999 after five years of SO_4_^2−^ treatment provide evidence that the elevation of superficial peat pore water MeHg by long-term SO_4_^2−^ deposition can be persistent and endure throughout the snow-free period. The plausibility of peat pore water just below the water table being the source of the MeHg in runoff from the study wetland was confirmed by the close correspondence between the MeHg concentrations in the superficial pore water of the LowS plots receiving ambient atmospheric deposition and the stream draining the study mire (Fig. 1A). Furthermore, the water table level is shown to be a major factor controlling the magnitude of the MeHg concentration enhancement in peat pore water on the sites with increased S deposition.

We believe that the persistent elevation of pore water MeHg concentrations and the connection to water table fluctuation can be explained according to the following conceptual model. Pore water MeHg is produced by SRB’s through the reduction of SO_4_^2−^ [12], [31], [54]. Sulphur deposited from the atmosphere to the mire surface is cycled between SO_4_^2−^ and hydrogensulphide (H_2_S), resulting from the changing redox conditions caused by the vertical movement of the GWL and the presence of SRB microbial communities. The GWL fluctuations depend on variations in evapotranspiration, precipitation and surface water runoff. One aspect of this conceptual model is a transient reduction of the recently deposited SO_4_^2−^ by the SRB’s with a concomitant transient Hg methylation [30], [35], [55]. The current study indicates that a chronic deposition of anthropogenic SO_4_^2−^ also results in a more long-term reduction/oxidation cycle of the previously deposited S which sustains elevated MeHg levels for weeks after the last precipitation input of SO_4_^2−^ to the mire. That long-term redox cycle is driven by the microbial decomposition of organic matter and changing redox-conditions. The possibility of strong internal cycling of S due to variations in redox conditions has been shown earlier for peatlands [38], [56], [57].

This conceptual “SRB” model is supported by the temporal variation in pore water MeHg found in the HighS plots that correlates significantly with the GWL over the season (Fig. 1). During one distinct period the pore water concentrations of MeHg in the HighS plots were close to the concentrations found in the plots receiving ambient levels of SO_4_^2−^. This period coincided with the lowest GWL and the highest pore water concentrations of SO_4_^2−^ measured at the HighS plots (Fig. 3). We interpret this temporal variation in MeHg as a response to the vertical distribution of the anaerobic heterotrophic bacteria delivering the carbon precursors used by sulphate reducing bacteria. The vertical distribution for other heterotrophic microorganisms in mires, e.g. methanogenic archaea and methanotrophic bacteria is related to the average growing season mire water table level and does not change directly in response to the actual GWL [58]–[61]. The methanogenic archaea, utilizing the same carbon and energy source as the SRB, has its maxima just below the average depth for the growing season water table level [59]–[61] indicating competitive exclusion or reduction of methanogens by dissimilatory SRB [61], [62]. Therefore, when the water table had declined to low levels as a result of the unusually long period of dry summer weather, SO_4_^2−^ reduction and concomitant Hg methylation did not occur fast enough to make use of all the SO_4_^2−^ that was available due to a smaller population of SRB. This is in contrast to the situation with higher more normal GWL when our sampling takes place at a level where the SRB community is apparently larger because it can completely exploit the available SO_4_^2–^rapidly enough to keep SO_4_^2−^ concentrations below detectable levels, c.f. Eriksson *et al*. [61].

A decrease in MeHg concentrations with depth (Fig. 2) also suggests that the methylation activity of the SRB’s decreases with depth, and that the effect of enhanced SO_4_^2−^ deposition on Hg methylation is highest within the area of regular groundwater fluctuations. This zone of regularly fluctuating water tables is also the zone that is likely to contribute most to runoff from this type of wetland [63], [64]. Altogether this indicates that SO_4_^2−^ reduction is restricted at depth. This would be expected and results mainly from the vertical distribution of high quality carbon substrate, acting as an electron donor to the SRBs, which is highest close to the mire surface. Thus, the abundance of SRB’s would be relatively lower at these greater depths. (Note that the absolute depth of pore water sampling relative to the mire surface varies with the water table).

While most of the data were consistent with the “SRB” hypothesis, there was one piece of evidence in this study that does not fit with this theory. That is the lack of correlation between solid phase S in the peat, and pore water MeHg (Table 1). The experimental additions of SO_4_^2−^ every 4 or 5 weeks during the snow-free period are expected to be reduced within hours or days after addition [30], and indeed SO_4_^2−^ was below detection limit for most of the study period, with the one exception during the period of low GWL from mid-August to mid-September. Therefore, according to the hypothesis, it is increased concentrations of S in the organic matter that should feed the persistent effect from the SO_4_^2−^ addition found in the HighS plots. Such a correlation between total S in the peat and elevated pore water MeHg levels (or even S additions) was not found in our study. However, in the study from 1997 [36] done on the same site, a positive correlation between pore water MeHg and S in the solid phase was found, supporting the hypothesis described above. We believe that more needs to be known about the cycling of S in the mire to devise measurements better suited to testing this aspect of the SRB hypothesis.

The factors which pore water MeHg was not related to are also important arguments for the SRB hypothesis. To begin with, there was no correlation between Hg_tot_ and MeHg. Such a lack of correlation between pore water MeHg and Hg_tot_ was also found in a variety of aqueous samples [65], [66].

High DOC and low pH [67]–[70] have been shown to correlate to increased MeHg concentrations in lake water and fish. Neither of these correlations are a direct outcome of the SRB hypothesis, and no such correlations between DOC and MeHg concentrations were found in this study of peat pore water that is a precursor to runoff. In late August, water was sampled from the lowest peat depth during the season, yielding DOC concentrations twice as high as on the other summer and autumn sampling occasions. This increase in DOC concentration was accompanied by only a very limited increase in MeHg in the HighS plots. The pH in the HighS plots was approximately 0.2–0.4 pH units higher than in the LowS plots at the beginning of the snow free season when the HighS plots had the greatest elevation of pore water MeHg relative to the LowS plots. The microbial reduction of SO_4_^2−^ consumes H^+^ [71] and theoretically, the complete reduction of the added SO_4_^2−^ would be sufficient to generate the higher pH in the HighS plots. Thus, neither acidification, DOC nor Hg_tot_ could account for the enhanced concentrations and temporal variation of MeHg after SO_4_^2−^ additions.

Another alternative explanation for the enhanced levels of pore water MeHg found in the HighS plots might be that the microbially produced MeHg sulphides which increase the partitioning of MeHg to the water phase [72], [73]. However, calculations based on the amount of added SO_4_^2−^ revealed that a negligible concentration of MeHg bound to peat organic matter would be exchanged due to the low pH in the peat.

### Conclusion

This study demonstrates that chronic deposition of SO_4_^2−^ to a boreal mire can result in large and persistent increases in the MeHg of peat pore water located near the surface of the water table. The increase in pore water MeHg caused by the addition of 17 kg S ha yr^−1^ beyond the ambient deposition of 3 kg ha^−1^ yr^−1^ varied strongly over the snow free season in conjunction with seasonal fluctuations of the water table. The conceptual model we present to explain the HighS effect on MeHg postulates that enhanced pore water MeHg concentrations are maintained by the cycling of S as a result of fluctuating redox conditions that stimulate, and presumably increase the SRB community in the immediate vicinity of the redox fluctuation associated with the fluctuating water table.

Since the peat pore water just below the water table can be an important source of MeHg in runoff from a wetland, we believe these results support the hypothesis that SO_4_^2−^ deposition has increased the contribution of peatlands to MeHg in downstream aquatic systems. This would mean that the increased deposition of SO_4_^2−^ in acid rain has contributed to the modern increase in the MeHg burdens of remote lakes hydrologically connected to peatlands, as has also been suggested by other studies [9], [36]. This would help explain why the catchment yields of MeHg from small boreal catchments are not well correlated to the atmospheric input of Hg [19], [74]. According to this “sulphur rain” hypothesis, the large catchment MeHg yields are enhanced by the stimulation of Hg methylation by SO_4_^2−^ in acid rain, rather than directly from the Hg deposition itself or as a consequence of surface water acidification.

The cycling of MeHg in catchments is complex, and involves numerous biogeochemical and physical controls, many of which remain to be satisfactorily quantified. The findings presented here are not likely to be applicable to all catchments, but if they are applicable to many high-latitude catchments containing peatlands or other areas of anoxic organic sediments, then they represent an important link in our understanding of catchment-scale Hg cycling with implications for Hg uptake by fish that extend across the boreal and sub-boreal zones of North America, Europe and Asia.
