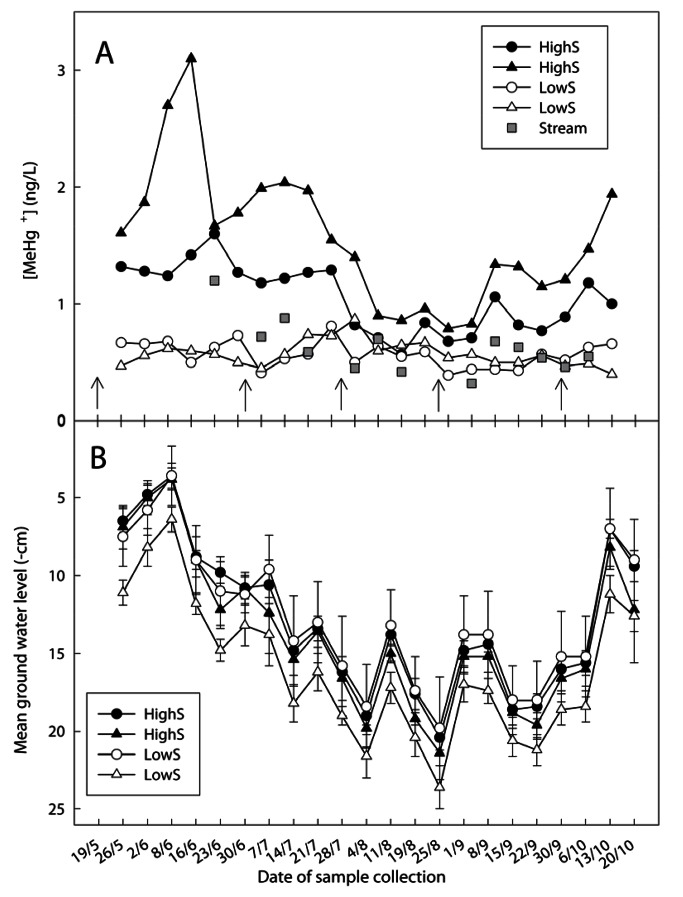# Correction: The Influence of Sulphate Deposition on the Seasonal Variation of Peat Pore Water Methyl Hg in a Boreal Mire

**DOI:** 10.1371/annotation/c81e29f5-0042-4155-bcba-19839ed573b3

**Published:** 2013-05-14

**Authors:** Inger Bergman, Kevin Bishop, Qiang Tu, Wolfgang Frech, Staffan Åkerblom, Mats Nilsson

There was an error in Figure 1. The correct version of the figure is available here: 

**Figure:**